# Plastic Wound Protector vs Surgical Gauze for Surgical Site Infection Reduction in Open GI Surgery: A Randomized Clinical Trial

**DOI:** 10.1001/jamasurg.2024.0765

**Published:** 2024-04-24

**Authors:** Nina Yoo, Ji Yeon Mun, Bong-Hyeon Kye, Chang Woo Kim, Jae Im Lee, Youn Young Park, Byung Mo Kang, Byung Kwan Park, Han Deok Kwak, Won-Kyung Kang, Sung Uk Bae, Heung-Kwon Oh, Youngki Hong, Hyung Jin Kim

**Affiliations:** 1 Department of Surgery, Seoul St Mary’s Hospital, College of Medicine, The Catholic University of Korea, Seoul, Korea; 2 Department of Surgery, St Vincent’s Hospital, College of Medicine, The Catholic University of Korea, Suwon, Korea; 3 Department of Surgery, Ajou University Hospital, Suwon, Korea; 4 Department of Surgery, Uijeongbu St Mary’s Hospital, College of Medicine, The Catholic University of Korea, Uijeongbu, Korea; 5 Department of Surgery, Kyung Hee University Hospital at Gangdong, Kyung Hee University College of Medicine, Seoul, Korea; 6 Department of Surgery, Chuncheon Sacred Heart Hospital, Hallym University College of Medicine, Chuncheon, Korea; 7 Department of Surgery, Chung-Ang University Hospital, Chung-Ang University College of Medicine, Seoul, Korea; 8 Department of Surgery, Chonnam National University Hospital, College of Medicine, Chonnam National University, Gwangju, Korea; 9 Department of Surgery, Yeouido St Mary’s Hospital, College of Medicine, The Catholic University of Korea, Seoul, Korea; 10 Department of Surgery, Keimyung University and Dongsan Medical Center, Daegu, Korea; 11 Department of Surgery, Seoul National University Bundang Hospital, Seongnam, Korea; 12 Department of Surgery, National Health Insurance Service, Ilsan Hospital, Goyang, Korea; 13 Department of Surgery, EunPyeong St Mary’s Hospital, College of Medicine, The Catholic University of Korea, Seoul, Korea

## Abstract

**Importance:**

Surgical site infections (SSIs) are prevalent hospital-acquired infections with significant patient impacts and global health care burdens. The World Health Organization recommends using wound protector devices in abdominal surgery as a preventive measure to lower the risk of SSIs despite limited evidence.

**Objective:**

To examine the efficacy of a dual-ring, plastic wound protector in lowering the SSI rate in open gastrointestinal (GI) surgery irrespective of intra-abdominal contamination levels.

**Design, Setting, and Participants:**

This multicenter, patient-blinded, parallel-arm randomized clinical trial was conducted from August 2017 to October 2022 at 13 hospitals in an academic setting. Patients undergoing open abdominal bowel surgery (eg, for bowel perforation) were eligible for inclusion.

**Intervention:**

Patients were randomized 1:1 to a dual-ring, plastic wound protector to protect the incision site of the abdominal wall (experimental group) or a conventional surgical gauze (control group).

**Main Outcomes and Measures:**

The primary end point was the rate of SSI within 30 days of open GI surgery.

**Results:**

A total of 458 patients were randomized; after 1 was excluded from the control group, 457 were included in the intention-to-treat analysis (mean [SD] age, 58.4 [12.1] years; 256 [56.0%] male; 341 [74.6%] with a clean-contaminated wound): 229 in the wound protector group and 228 in the surgical gauze group. The overall SSI rate in the intention-to-treat analysis was 15.7% (72 of 458 patients). The SSI rate for the wound protector was 10.9% (25 of 229 patients) compared with 20.5% (47 of 229 patients) with surgical gauze. The wound protector significantly reduced the risk of SSI, with a relative risk reduction (RRR) of 46.81% (95% CI, 16.64%-66.06%). The wound protector significantly decreased the SSI rate for clean-contaminated wounds (RRR, 43.75%; 95% CI, 3.75%-67.13%), particularly for superficial SSIs (RRR, 42.50%; 95% CI, 7.16%-64.39%). Length of hospital stay was similar in both groups (mean [SD], 15.2 [10.5] vs 15.3 [10.2] days), as were the overall postoperative complication rates (20.1% vs 18.8%).

**Conclusions and Relevance:**

This randomized clinical trial found a significant reduction in SSI rates when a plastic wound protector was used during open GI surgery compared with surgical gaze, supporting the World Health Organization recommendation for use of wound protector devices in abdominal surgery.

**Trial Registration:**

ClinicalTrials.gov Identifier: NCT03170843

## Key Points

**Question:**

Does a plastic wound protector lower surgical site infection (SSI) rates compared with surgical gauze in open gastrointestinal surgeries?

**Findings:**

In this randomized clinical trial of 458 patients, the wound protector decreased SSI risk by 46.8% across bowel surgeries, with a 43.8% decrease for clean-contaminated wounds and 42.5% for superficial SSIs, compared with surgical gauze. Its effect on contaminated wounds was less certain.

**Meaning:**

Plastic wound protectors are effective in reducing SSIs in open gastrointestinal surgeries.

## Teaser

This randomized clinical trial examines whether a plastic wound protector lowers surgical site infection rates compared with surgical gauze in open gastrointestinal (GI) surgeries.

## Introduction

Surgical site infection (SSI) is a common postoperative complication in patients undergoing general abdominal surgery. It is associated with a significant burden for health care practitioners and patients, with extra medical expenses, time, and human resources.^1^ Global guidelines and recommendations for preventing SSI suggest regulating risk factors and applying preventive measures in the preoperative, intraoperative, and postoperative periods.^2,3,4^ To provide uniform and clear instructions on SSI prevention, the World Health Organization (WHO) developed evidence-based recommendations for preoperative, intraoperative, and postoperative periods.^5,6^ In the intraoperative period, the use of a wound protector device is recommended to reduce the rate of SSI in clean-contaminated, contaminated, and dirty abdominal surgical procedures.^6^

However, the WHO panel^6^ suggested its use with conditional recommendation with a very low quality of evidence. A meta-analysis based on 10 randomized clinical trials and 1 prospective clinical trial indicated that use of a wound protector device was associated with a lower risk of SSI compared with conventional wound protection (odds ratio, 0.42; 95% CI, 0.28-0.62).^6^ However, the studies included in the meta-analysis consisted of a heterogeneous population, such as patients undergoing cesarean delivery, those with fecal peritonitis, or those with hepatobiliary surgery. Moreover, data on patients who present with contaminated or dirty wounds are scarce. Therefore, the effectiveness of reducing SSI for surgical procedures dealing with contaminated or dirty wounds has not been shown, to our knowledge. Additional data with a comparable study population and control of various confounding factors are necessary to provide strong evidence supporting the recommendation. Therefore, to provide high-quality evidence, this study evaluated the effectiveness of a plastic wound protector in reducing the rate of SSI for patients undergoing open abdominal gastrointestinal (GI) surgery.

## Methods

### Study Design and Participating Centers

This patient-blinded, multicentered, randomized clinical trial compared a protective plastic dual-ring wound retractor with conventional surgical gauze for incisional wound protection in open abdominal GI surgery (NCT03170843; trial protocol in Supplement 1). From August 2017 to October 2022, this study was conducted and analyzed following the Consolidated Standards of Reporting Trials (CONSORT) guideline.^7^ The study protocol was publicly opened after trial initiation for further recruitment and to assist participating investigators and study coordinators in conducting the study as planned.^8^ A total of 13 referral hospitals in an academic setting in South Korea participated. There have been no major changes to the trial methods since the beginning. The institutional review board at each participating center reviewed the trial protocol and informed consent document and granted ethical approval. All participants provided written informed consent.

### Participants

Patients undergoing open abdominal GI surgery were eligible for inclusion. Inclusion criteria were the following: (1) age of 18 to 75 years, (2) undergoing elective or emergent open abdominal surgery, and (3) undergoing surgery on the stomach, small intestine, or colon and rectum. Patients were excluded if they exhibited any of the following: (1) presence of concurrent infection in the abdominal wall; (2) open conversion from laparoscopic surgery; (3) presence of poor nutritional status, indicated by a Nutritional Risk Screening^9^ 2002 score of 3 or greater; (4) undergoing combined hepatobiliopancreatic surgery; (5) pregnancy or breastfeeding; and (6) moderate to severe immunosuppression state, defined as previous organ or bone marrow transplant, concurrent corticosteroid administration (>10 mg prednisolone daily or an equivalent dose of any other corticosteroid), or concurrent administration of other immunosuppressive or chemotherapeutic agents within the 2 weeks before trial intervention.

### Surgical Interventions

An open laparotomy was made once a patient was administered general anesthesia. A dual-ring wound protector (O Trac; Asung Medical Inc) was applied to the incision site in the patients in the experimental group (eFigure 1 in Supplement 2). The patients in the control group had their incision site covered with conventional surgical gauze (eFigure 2 in Supplement 2). The wound protector and the surgical gauze were left in situ during the entire operation and immediately removed just before closing the abdominal wall. The details of preoperative, intraoperative surgical, and postoperative procedures followed the policy of an individual surgeon and institutional infection control policy at each center. Board-certified general surgeons performed all surgical procedures in an academic setting.

All investigators were mandated to adhere to the SSI prevention bundle, particularly for elective colorectal surgeries.^6,10^ This comprehensive protocol includes mechanical bowel preparation, prophylactic antibiotic administration, surgical field antisepsis, and the maintenance of intraoperative normothermia. All participating centers used disposable surgical gloves and gowns. However, the replacement of surgical gowns and gloves was at the discretion of the operating surgeon. Wound irrigation was performed using normal saline rather than an antibiotic solution. Additionally, no specific wound dressing type was mandated; selection was based on each investigator’s preference.

### Study Outcomes

The primary outcome was the difference in rates of SSIs between 2 groups: one using the plastic wound protector (experimental group) and the other using conventional surgical gauze (control group). Surgical site infections were defined by the diagnostic criteria suggested by the US Centers for Disease Control and Prevention within 30 days after surgery and classified as superficial incisional, deep incisional, and organ or space.^11^ The secondary outcome was to compare the length of postoperative hospital stay and the rate of surgical complications other than SSI in the 2 groups. The postoperative surgical complications were classified according to the modified Clavien-Dindo classification.^12^ Other secondary outcomes included the hospital readmission rate; however, patients diagnosed with a malignant disease after surgery required readmission for chemotherapy postoperatively. Therefore, the hospital readmission rate was not counted as the secondary outcome.

### Data Collection

A web-based electronic case reporting form (eCRF) was used to record data on the patients.^13^ Patient baseline characteristics, the parameters for the surgical procedure, and the perioperative laboratory parameters were reported in a timely manner (detailed parameters are given in Supplement 1). Each surgeon responsible for enrolled patients evaluated the patient’s surgical wound at postoperative weeks 1, 2 to 3, and 4 to 5. In cases in which patients did not adhere to office visits, a telephone interview was conducted to identify any symptoms or signs of infection or inflammation in the surgical site. A photograph of the wound was taken at each office visit and uploaded in the eCRF. If SSI was detected, its classification and the postoperative date of diagnosis were recorded. Confirmation of SSI was made using the photograph by 2 others who were not involved in the clinical trial. Postoperative complications categorized by the modified Clavien-Dindo classification and postoperative length of hospital stay were documented. All the data were entered in the eCRF by an investigator or research coordinator at each center.

### Power Calculation

This clinical trial investigated the superiority of a plastic wound retractor in reducing the rate of SSIs compared with a conventional surgical gauze. Initially, the ratio of operations with clean or clean-contaminated, contaminated, and dirty infected wounds was estimated to be 20%:40%:40% based on a review of published data^14,15,16,17,18^ and our experience. As the SSI incidence was reported as 10% for clean or clean-contaminated, 25% for contaminated, and 40% for dirty infected wounds, the incidence of SSI for the control group was 28%, and the incidence of SSI was expected to be reduced by a range of 17% to 40% in the experimental group.^14,15,16,17,18^ A sample size of 434 participants was determined to achieve a study power of 80% with 2-sided 95% CIs. Considering a dropout rate up to 5%, a total of 458 patients, 229 patients in each group, were anticipated to participate in the study.

### Randomization and Blinding

Patients were enrolled by treating surgeons or permitted research personnel. A biostatistician predefined the group allocation and randomization sequence. Patients were randomized 1:1 to the experimental or control group. A permuted block randomization with the size of 2 or 4 was applied. On the successful screening, the patient was stratified according to the anticipated category of wound contamination, with 2 separately powered strata: one with clean contaminated wounds and the other with contaminated or dirty infected wounds. A web-based patient registry^13^ was used to allocate each patient before the beginning of the operation, which provided adequate concealment for the allocation sequence. Although participating surgeons were not blinded to the allocated treatment, the patients were blinded to the trial intervention. Once a patient was identified and agreed to participate in the trial, the patient was screened for fitness to participate. The data manager was also blinded due to a lack of access to the trial intervention and the randomization.

### Statistical Analysis

The statistical analysis was performed by an independent statistician from The Catholic Medical Center (Seoul, South Korea). The result was analyzed for the intention-to-treat (ITT) population and the per-protocol (PP) population. The rate of 30-day postoperative SSI was evaluated in all patients and analyzed according to the wound classification: superficial incisional, deep incisional, and organ or space SSIs. Pearson χ^2^ test or Fisher exact test was used to analyze nominal data. The *t* test and the Wilcoxon rank sum test were used for continuous variables. *P* values were also calculated from the Cochran-Mantel-Haenszel test, stratified by wound type, the randomization stratification factor. The difference was constructed for the control minus the wound protector group, and the 95% CIs were constructed using the Wald method. Sensitivity analysis was performed for participants with organ-space infection who were excluded from the trial. The statistical analysis was conducted using SAS, version 9.4 (SAS Institute Inc). Two-sided *P* < .05 was considered significant.

## Results

From August 2017 to October 2022, 458 patients were enrolled and randomly assigned to the experimental group or the control group (Figure). Initially, 229 patients were allocated to each group as the ITT population. However, after randomization, 1 patient in the control group was found to have violated a screening protocol, leading to an ITT population of 457 patients (201 [44.0%] female and 256 [56.0%] male): 229 in the wound protector group and 228 in the control group. The mean (SD) age was 58.4 (12.1) years, with a median age of 60.0 years (IQR, 52.0-68.0 years). Subsequently, 19 patients from the wound protector group and 26 patients from the control group were excluded from the study, leaving 210 and 202 patients in the PP analysis for the experimental and control groups, respectively. The reasons for these exclusions are detailed in the CONSORT diagram (Figure).

**Figure.**
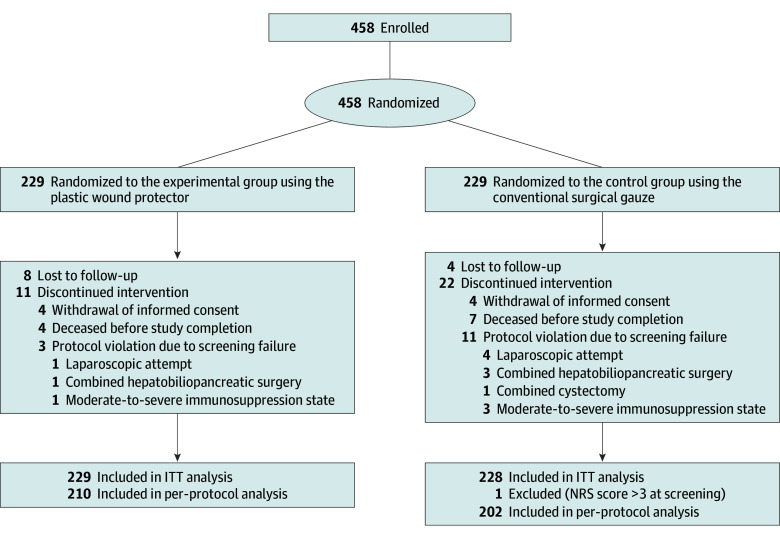
CONSORT Flowchart ITT indicates intention-to-treat; NRS, Nutritional Risk Screening.

Table 1 shows the baseline characteristics of patients in the ITT and PP populations. In a total of 457 patients, 341 (74.6%) had a clean-contaminated wound, with the remaining 116 (25.4%) having a contaminated or dirty infected wound. Overall, there was no significant difference between the 2 groups except in the PP analysis, in which body mass index (calculated as weight in kilograms divided by height in meters squared) was slightly higher in the control group than in the wound protector group (mean [SD], 23.8 [3.8] vs 23.0 [3.6]; *P* = .04).

**Table 1.** Baseline Patient Characteristics^a^

Characteristic	Intention-to-treat population			Per-protocol population		
	Total (n = 457)	Wound protector (n = 229)	Gauze (n = 228)	Total (n = 412)	Wound protector (n = 210)	Gauze (n = 202)
Wound type						
Clean-contaminated	341 (74.6)	171 (74.7)	170 (74.6)	306 (74.3)	155 (73.8)	151 (74.8)
Contaminated or dirty, infected	116 (25.4)	58 (25.3)	58 (25.4)	106 (25.7)	55 (26.2)	51 (25.2)
Sex						
Female	201 (44.0)	100 (43.7)	101 (44.3)	185 (44.9)	92 (43.8)	93 (46.0)
Male	256 (56.0)	129 (56.3)	127 (55.7)	227 (55.1)	118 (56.2)	109 (54.0)
Age, y						
Mean (SD)	58.4 (12.1)	58.4 (12.2)	58.3 (12.0)	58.2 (12.1)	58.5 (12.2)	58.0 (12.1)
Median (IQR)	60.0 (52.0-68.0)	60.0 (52.0-68.0)	60.0 (52.0-67.5)	60.0 (52.0-68.0)	60.0 (52.0-68.0)	60.0 (51.0-67.0)
Age-adjusted preoperative NRS-2002 score^b^						
0	235 (51.4)	116 (50.7)	119 (52.2)	213 (51.7)	106 (50.5)	107 (53.0)
1	111 (24.3)	61 (26.6)	50 (21.9)	99 (24.0)	55 (26.2)	44 (21.8)
2	111 (24.3)	52 (22.7)	59 (25.9)	100 (24.3)	49 (23.3)	51 (25.2)
BMI						
Mean (SD)	23.3 (3.7)	23.0 (3.6)	23.6 (3.8)	23.4 (3.8)	23.0 (3.6)	23.8 (3.8)
Median (IQR)	23.1 (20.7-25.3)	22.7 (20.5-25.1)	23.5 (21.3-25.6)	23.2 (20.8-25.4)	22.7 (20.5-25.1)	23.6 (21.5-25.6)
ASA class						
I or II	374 (81.8)	186 (81.2)	188 (82.5)	350 (85.0)	177 (84.3)	173 (85.6)
III or IV	75 (16.4)	39 (17.0)	36 (15.8)	62 (15.0)	33 (15.7)	29 (14.4)
History of diabetes						
Yes	80 (17.5)	41 (17.9)	39 (17.1)	72 (17.5)	37 (17.6)	35 (17.3)
No	369 (80.7)	184 (80.3)	185 (81.1)	340 (82.5)	173 (82.4)	167 (82.7)
Smoking habit						
Yes	67 (14.7)	36 (15.7)	31 (13.6)	55 (13.3)	31 (14.8)	24 (11.9)
No	382 (83.6)	189 (82.5)	193 (84.6)	357 (86.7)	179 (85.2)	178 (88.1)
Alcohol consumption						
Yes	110 (24.1)	58 (25.3)	52 (22.8)	99 (24.0)	53 (25.2)	46 (22.8)
No	339 (74.2)	167 (72.9)	172 (75.4)	313 (76.0)	157 (74.8)	156 (77.2)
Necessity of postoperative ICU care						
Yes	50 (10.9)	23 (10.0)	27 (11.8)	45 (10.9)	22 (10.5)	23 (11.4)
No	399 (87.3)	202 (88.2)	197 (86.4)	367 (89.1)	188 (89.5)	179 (88.6)
History of chemotherapy						
Yes	91 (19.9)	45 (19.7)	46 (20.2)	86 (20.9)	44 (21.0)	42 (20.8)
No	358 (78.3)	180 (78.6)	178 (78.1)	326 (79.1)	166 (79.0)	160 (79.2)
History of radiotherapy						
Yes	30 (6.6)	16 (7.0)	14 (6.1)	27 (6.6)	16 (7.6)	11 (5.4)
No	419 (91.7)	209 (91.3)	210 (92.1)	385 (93.4)	194 (92.4)	191 (94.6)
History of abdominal surgery						
Yes	205 (44.9)	101 (44.1)	104 (45.6)	194 (47.1)	97 (46.2)	97 (48.0)
No	244 (53.4)	124 (54.1)	120 (52.6)	218 (52.9)	113 (53.8)	105 (52.0)
Corticosteroid use						
Yes	9 (2.0)	2 (0.9)	7 (3.1)	7 (1.7)	2 (1.0)	5 (2.5)
No	440 (96.3)	223 (97.4)	217 (95.2)	405 (98.3)	208 (99.0)	197 (97.5)
Immunosuppressant use						
Yes	5 (1.1)	3 (1.3)	2 (0.9)	5 (1.2)	3 (1.4)	2 (1.0)
No	444 (97.2)	222 (96.9)	222 (97.4)	407 (98.8)	207 (98.6)	200 (99.0)
Antiplatelet or anticoagulant use						
Yes	30 (6.6)	16 (7.1)	14 (6.1)	26 (6.3)	15 (7.1)	11 (5.4)
No	419 (91.7)	209 (91.3)	210 (92.1)	386 (93.7)	195 (92.9)	191 (94.6)
GI cancer history						
Yes	108 (23.6)	56 (24.5)	52 (22.8)	98 (23.8)	53 (25.2)	45 (22.3)
No	341 (74.6)	169 (73.8)	172 (75.4)	314 (76.2)	157 (74.8)	157 (77.7)

Abbreviations: ASA, American Society of Anesthesiologists; BMI, body mass index (calculated as weight in kilograms divided by height in meters squared); GI, gastrointestinal; ICU, intensive care unit; NRS-2002, Nutritional Risk Screening 2002.

^a^ Data are given as number (percentage) of patients, unless otherwise indicated.

^b^ A score of 0 indicates a normal nutritional status; 1, mild impairment of nutritional status; and 2, moderate impairment of nutritional status.

Table 2 outlines the preoperative and intraoperative characteristics, including interventions. Emergency surgeries accounted for 161 (36.7%) in the ITT population and 153 (37.1%) in the PP population. The most common surgical site was the colorectum (324 patients [70.9%] in the ITT population and 296 [71.8%] in the PP population). Contaminated or dirty infected wounds were present in 124 (27.1%) in the ITT population and 117 (28.4%) in the PP population. Preoperative and surgical factors were largely comparable between the groups. Notably, in the PP group, the control group’s incision length was significantly longer than that in the wound protector group (mean [SD], 20.3 [5.8] vs 19.1 [5.1] cm; *P* = .03).

**Table 2.** Preoperative and Intraoperative Characteristics^a^

Characteristic	Intention-to-treat population			Per-protocol population		
	Total (n = 457)	Wound protector (n = 229)	Gauze (n = 228)	Total (n = 412)	Wound protector (n = 210)	Gauze (n = 202)
Surgery timing						
Elective	285 (62.4)	140 (61.1)	145 (63.6)	259 (62.9)	130 (61.9)	129 (63.9)
Emergency	161 (35.2)	84 (36.7)	77 (33.8)	153 (37.1)	80 (38.1)	73 (36.1)
Surgical site						
Stomach	14 (3.1)	4 (1.7)	10 (4.4)	12 (2.9)	3 (1.4)	9 (4.5)
Small bowel	108 (23.6)	54 (23.6)	54 (23.7)	104 (25.2)	52 (24.8)	52 (25.7)
Colorectal	324 (70.9)	166 (72.5)	158 (69.3)	296 (71.8)	155 (73.8)	141 (69.8)
Trauma-related surgery						
Yes	7 (1.5)	3 (1.3)	4 (1.8)	259 (62.9)	130 (61.9)	129 (63.9)
No	439 (96.1)	221 (96.5)	218 (95.6)	153 (37.1)	80 (38.1)	73 (36.1)
Type of skin preparation						
Ethanol	6 (1.3)	3 (1.3)	3 (1.3)	5 (1.5)	3 (1.8)	2 (1.3)
Isopropyl alcohol	2 (0.4)	1 (0.4)	1 (0.4)	2 (0.6)	1 (0.6)	1 (0.7)
Aqueous povidone	259 (56.7)	139 (60.7)	120 (52.6)	242 (74.7)	132 (77.2)	110 (71.9)
Chlorohexidine	64 (14.0)	34 (14.8)	30 (13.2)	57 (17.6)	28 (16.4)	29 (19.0)
>2 Substances	18 (3.9)	7 (3.1)	11 (4.8)	18 (5.6)	7 (4.1)	11 (7.2)
Degree of intraperitoneal contamination						
Clean-contaminated	322 (70.5)	164 (71.6)	158 (69.3)	295 (71.6)	153 (72.9)	142 (70.3)
Contaminated or dirty, infected	124 (27.1)	60 (26.2)	64 (28.1)	117 (28.4)	57 (27.1)	60 (29.7)
Use of antibiotics						
Therapeutic	233 (50.8)	118 (51.5)	115 (50.4)	219 (53.2)	111 (52.9)	108 (53.5)
Prophylactic	212 (46.4)	106 (46.3)	106 (46.5)	193 (46.8)	99 (47.1)	94 (46.5)
Total surgery time, min						
Mean (SD)	169.2 (88.0)	168.7 (86.5)	169.7 (89.7)	170.0 (88.2)	170.0 (87.7)	170.0 (88.9)
Median (IQR)	150.0 (107.0-210.0)	145.0 (110.0-207.5)	153.0 (105.0-215.0)	150.0 (105.5-215.0)	145.5 (110.0-210.0)	154.5 (105.0-215.0)
Bowel anastomosis						
Yes	356 (77.9)	180 (78.6)	176 (77.2)	329 (79.9)	169 (80.5)	160 (79.2)
No	90 (19.7)	44 (19.2)	46 (20.2)	83 (20.1)	41 (19.5)	42 (20.8)
Colostomy formation						
Yes	82 (17.9)	41 (17.9)	41 (18.0)	75 (18.2)	38 (18.1)	37 (18.3)
No	364 (79.6)	183 (79.9)	181 (79.4)	337 (81.8)	172 (81.9)	165 (81.7)
Skin suture material						
Nylon	102 (22.3)	48 (21.0)	54 (23.7)	89 (21.6)	42 (20.0)	47 (23.3)
Vicryl	22 (4.8)	14 (6.1)	8 (3.5)	20 (4.9)	13 (6.2)	7 (3.5)
Skin stapler	240 (52.5)	124 (54.1)	116 (50.9)	230 (55.8)	121 (57.6)	109 (54.0)
>2 Substances	82 (17.9)	38 (16.6)	44 (19.8)	73 (17.7)	34 (16.2)	39 (19.3)
Incision length, cm						
Mean (SD)	19.7 (5.4)	19.2 (5.0)	20.3 (5.7)	19.7 (5.4)	19.1 (5.1)	20.3 (5.8)
Median (IQR)	20.0 (16.0-22.5)	19.0 (16.0-22.0)	20.0 (17.0-23.0)	20.0 (16.0-22.0)	19.0 (16.0-22.0)	20.0 (17.0-23.0)
Use of drainage system on the superficial wound						
Yes	145 (31.7)	76 (33.2)	69 (30.3)	136 (33.0)	71 (33.8)	65 (32.2)
No	301 (65.9)	148 (64.6)	153 (67.1)	276 (67.0)	139 (66.2)	137 (67.8)
Body temperature during surgery, °C						
Mean (SD)	36.4 (0.6)	36.4 (0.6)	36.4 (0.5)	36.4 (0.5)	36.4 (0.6)	36.4 (0.5)
Median (IQR)	36.4 (36.1-36.7)	36.3 (36.1-36.6)	36.4 (36.2-36.7)	36.4 (36.1-36.7)	36.3 (36.1-36.6)	36.4 (36.2-36.7)

^a^ Data are given as number (percentage) of patients, unless otherwise indicated.

Table 3 displays the SSI rates. The rate was 10.9% (25 of 229 patients) for the wound protector group and 20.5% (47 of 229 patients) for the control group, with an overall rate of 15.7% (72 of 458 patients). The wound protector achieved a statistically significant 46.81% relative risk reduction (95% CI, 16.64%-66.06%; *P* = .005). The wound protector significantly decreased the SSI rate for clean-contaminated wounds (relative risk reduction, 43.75%; 95% CI, 3.75%-67.13%), particularly for superficial SSIs (relative risk reduction, 42.50%; 95% CI, 7.16%-64.39%). Subgroup analysis showed consistent efficacy of the wound protector in reducing superficial SSIs compared with conventional gauze. Sensitivity analysis also indicated a significant reduction in superficial SSIs with the wound protector (eTable 1 in Supplement 2). No significant difference was observed in the length of postoperative hospital stay between the groups, with a mean (SD) of 15.2 (10.5) days for the wound protector group and 15.3 (10.2) days for the control group (*P* = .69). Postoperative complications occurred in 89 of 458 patients (19.4%), with no significant difference between the groups: 46 of 229 (20.1%) in the wound protector group vs 43 of 229 (18.8%) in the control group (*P* = .41). The Clavien-Dindo classification indicated a similar severity distribution of postoperative morbidity in both groups (eTable 2 in Supplement 2).

**Table 3.** Rates of SSI

Outcome	Patients with SSI, No. (%)			Difference, percentage points (95% CI)	RRR, % (95% CI)	*P* value	*P* value^a^
	Total	Wound protector	Gauze				
**Intention-to-treat analysis**							
Patients, No.	458	229	229	NA	NA	NA	NA
Any SSI	72 (15.7)	25 (10.9)	47 (20.5)	9.61 (3.00 to 16.22)	46.81 (16.64 to 66.06)	.005	.005
Wound type							
Clean-contaminated (n = 342)	50 (14.6)	18 (10.5)	32 (18.7)	8.19 (0.75 to 15.63)	43.75 (3.75 to 67.13)	.03	NA
Contaminated or dirty, infected (n = 116)	22 (19.0)	7 (12.1)	15 (25.9)	13.79 (−0.25 to 27.84)	53.33 (−5.96 to 79.45)	.06	NA
SSI type							
Superficial	63 (13.8)	23 (10.0)	40 (17.5)	7.42 (1.15 to 13.70)	42.50 (7.16 to 64.39)	.02	NA
Deep	7 (1.5)	3 (1.3)	4 (1.7)	0.44 (−1.81 to 2.68)	25.00 (−231.35 to 83.02)	>.99	NA
Organ-space	4 (0.9)	0	4 (1.7)	1.75 (0.05 to 3.44)	100 (NA to 100)	.12	NA
**Per-protocol analysis**							
Patients, No.	412	210	202	NA	NA	NA	NA
Any SSI	69 (16.7)	24 (11.4)	45 (22.3)	10.85 (3.68 to 18.02)	48.70 (19.04 to 67.49)	.003	.003
Wound type							
Clean-contaminated (n = 306)	47 (15.4)	17 (11.0)	30 (19.9)	8.90 (0.86 to 16.94)	44.80 (4.20 to 68.19)	.03	NA
Contaminated or dirty, infected (n = 106)	22 (20.8)	7 (12.7)	15 (29.4)	16.68 (1.39 to 31.98)	56.73 (2.51 to 80.79)	.03	NA
SSI type							
Superficial	60 (14.6)	22 (10.5)	38 (18.8)	8.34 (1.54 to 15.13)	44.31 (9.26 to 65.82)	.02	NA
Deep	7 (1.7)	3 (1.4)	4 (2.0)	0.55 (−1.95 to 3.06)	27.86 (−218.33 to 83.65)	.72	NA
Organ-space	4 (1.0)	0	4 (2.0)	1.98 (0.06 to 3.90)	100 (NA to 100)	.06	NA

Abbreviations: NA, not available; RRR, relative risk reduction; SSI, surgical site infection.

^a^ *P* value from the Cochran-Mantel-Haenszel test, stratified by type of wound (randomization stratification factor).

## Discussion

This randomized clinical trial found that using a plastic wound protector during open abdominal GI surgery reduced the occurrence of SSI by 46.81% compared with using conventional surgical gauze. This is consistent with previous research that showed a reduced odds of SSI associated with the use of a dual-ring wound protector (odds ratio, 0.44; 95% CI, 0.35-0.56).^19^ Subgroup analyses further confirmed the consistent risk-reduction effect of the wound protector for both clean-contaminated and contaminated wounds. Overall, the evidence from this study robustly supports the efficacy of plastic wound protectors in preventing postoperative SSIs by shielding the incision site from bacterial contamination.

The overall SSI rate observed in this study was 15.7%, aligning with prior reports of SSI incidence for bowel, colon, and rectum procedures.^20^ This rate is within the expected 14% to 25% range for SSIs in open laparotomy for colon surgery and is consistent with the increase in SSI rates to 28% when contamination is present.^21,22^ Notably, the SSI rate in the wound protector group was 10.9%, which was half the rate in the control group using gauze. The substantial decrease in SSIs can also be credited to the meticulous application and observance of preventive practices, such as skin preparation, prophylactic antibiotic use, and maintenance of normothermia.

Based on literature on multiple risk factor analysis and preventive measures lowering SSI rates,^1,10^ guidelines for SSI prevention have been formulated and are broadly implemented by medical facilities and staff.^3,4,5,6,23^ In Korea, the Korean Nosocomial Infections Surveillance System, initiated in 2006, has been instrumental in promoting the adoption of these SSI prevention strategies.^24^ Additionally, the Korean Disease Control and Prevention Agency and the Korean Surgical Infection Society provide ongoing education on these protocols.^25^ It is reasonable to consider that such initiatives, coupled with the use of a wound protector acting as a physical barrier to bacterial contamination, could markedly decrease the rate of superficial SSIs.

In addition to wound protection, a plastic wound protector also enhances surgical field visibility by retracting the incision site. Despite the rise of minimally invasive techniques that contribute to the reduction of SSI,^26,27,28^ many patients still require open GI surgery.^29^ A self-retaining plastic wound retractor, designed for smaller incisions but wider operative views, was shown to significantly shorten incision lengths in 2 studies.^30,31^ Thus, the plastic wound protector not only serves as a physical barrier against postoperative SSIs but also may aid surgeons by functioning as an intraoperative retractor.

As expected, a plastic wound protector could not prevent the deep wound or organ-space infections in this study. A patient who had upper GI surgery developed an abscess in the retroperitoneum, while 3 patients who underwent colorectal procedures experienced anastomotic leaks. Anastomotic leakage is largely influenced by tension, blood supply, and bacterial infection.^32,33,34^ Ischemia in the site of surgery can also contribute to or worsen intraperitoneal abscesses.^35^ Therefore, patient factors, such as hemodynamic stability or degree of contamination, may be associated with the development of organ-space infection. Thoroughly washing the contaminated abdomen may help in reducing the chances of intra-abdominal abscesses. Fundamentally, due to the complex nature of factors contributing to organ-space infection, it is beyond the control of a plastic wound protector.

In this study, the incidence of deep wound infections was comparable between the 2 groups, with rates of 1.3% and 1.7%, respectively. Deep wound infections, unlike superficial SSIs that stem from exudate in the subcutaneous space, involve the fascial and muscle layers, which are typically closed during surgery to prevent evisceration, barring situations like acute compartment syndrome. At the surgeon’s discretion, a closed drainage system was implemented selectively to control exudate and potentially lower the risk of deep infections. However, factors beyond bacterial contamination also played a role; the healing of deep fascial wounds is contingent on maintaining adequate tension, blood flow, and oxygenation.^36^ Consequently, the development of deep wound infections is a multifaceted issue dependent on bacterial load, mechanical stress, and tissue perfusion. While a wound protector can limit bacterial contact with the wound, effectively preventing deep infections also necessitates enhancing overall patient condition to ensure optimal tissue healing.

### Strengths and Limitations

A strength of this study is that the controlled factors included preoperative nutritional status and wound class. Before enrollment, each patient was assessed for nutritional status along with preoperative medical conditions that might interfere with the wound healing process. By controlling crucial factors associated with postoperative SSI development, we could investigate the dominant effect of a plastic wound protector on SSI risk. Another strength is that the study population included patients in an emergency setting with purulent or fecal peritonitis. Including only bowel surgery, this study solely evaluated the effect of a wound protector on lowering the SSI risk particularly for colorectal surgery.

This study has limitations that should be addressed. First, the study did not identify the readmission rate for SSI, making it difficult to accurately estimate cost-effectiveness. However, a postdischarge surveillance program using telephone calls was implemented to closely monitor patients and reduce missed diagnoses of SSI, ensuring reliable data on the SSI rate. Second, the cost-effectiveness of a plastic wound protector is challenging to speculate. While it effectively functions as a retractor and reduces the SSI rate, its environmental impact and resource consumption have not been investigated. Plastic wound protectors contribute to medical waste and environmental degradation. Developing a biodegradable alternative may be ideal but could be costlier and impact cost-effectiveness negatively.^37^ Alternatively, implementing better recycling programs for medical plastics could reduce pollution risks. However, when evaluating cost-effectiveness, it is important to consider not only health care costs but also the device’s environmental impact and interpret the findings cautiously.

## Conclusion

This randomized clinical trial demonstrated that plastic wound protectors were effective in reducing the incidence of SSIs in open abdominal GI surgeries compared with traditional surgical gauze. Despite the challenges of assessing the cost-effectiveness and environmental impact of wound protectors, the clinical benefits are evident. Innovative efforts should concentrate on making these devices more environmentally sustainable while maintaining their effectiveness in infection prevention, prioritizing patient safety alongside ecological responsibility.

## Notes

Supplement 1. Trial Protocol

Supplement 2. **eFigure 1.** The Dual-Ring Wound Protector (O Trac, Asung Medical, South Korea) Applied to the Incision Site in the Patients of the Experimental Group **eFigure 2.** The Conventional Surgical Gauze Used to Cover the Incision Site in Patients in the Control Group **eTable 1.** Sensitivity Analysis for the Rates of Surgical Site Infection **eTable 2.** The Postoperative Complication Rates and Distribution of Complications by the Clavien-Dindo Classification

Supplement 3. Data Sharing Statement
