# Supplementary material for: Plastic Wound Protector vs Surgical Gauze for Surgical Site Infection Reduction in Open GI Surgery: A Randomized Clinical Trial
Source: JAMA Surg. 2024 Apr 24;159(7):737–46. doi: 10.1001/jamasurg.2024.0765 (PMC11044008; doi:10.1001/jamasurg.2024.0765)
Supplement: Supplement 1. — Trial Protocol [file jamasurg-e240765-s001.pdf]

Protocol

Executive summary

Title: Circular pOlyethylene drape in preVEntion of suRgical site infection (COVER trial): study protocol for a randomized controlled trial

Investigational product: O Trac, Asung Medical (a double-ring type of sterile, cylindrical wound protector consisting of inner and outer rings with a polyethylene sheath)

Protocol number: NCT#03170843

Study design: A prospective, multicenter, patient-blinded, randomized controlled trial with two parallel comparison arms.

Number of sites: Up to 13 sites of secondary or tertiary hospitals in South Korea

Number of subjects: A total of 458 patients undergoing open laparotomy, either elective or emergent, for gastrointestinal tract surgery

Study population: Male and female patients between the ages of 18 and 75 years requiring gastrointestinal surgery.

Study Criteria:

Inclusion criteria

1) Between the ages of 18 and 75 years
2) Undergoing elective or emergent open abdominal surgery
3) Undergoing surgery on the stomach, small intestine or colon and rectum

Exclusion criteria

1) Presence of concurrent infection in the abdominal wall
2) Open conversion from laparoscopic surgery
3) Presence of poor nutritional status indicated by a nutrition risk screening (NRS) 2002 score greater than 3
4) Undergoing combined hepatobiliopancreatic surgery
5) Pregnancy or breast feeding
6) Moderate to severe immunosuppression state (defined as previous organ or bone marrow transplantation, concurrent steroid administration of more than 10mg prednisolone daily or an equivalent dose of any other steroid, concurrent administration of other immunosuppressive or chemotherapeutic agents within the last 2 weeks prior to trial intervention)

Interventions: The incision site was covered by a circular polyethylene drape in the experimental group and the conventional surgical dressing gauze in the control group during the surgery

Study objective: To determine whether the device helps to reduce the overall rate of surgical site infection, SSI.

Study Endpoints:

Primary outcome

- The rate of SSI, defined by the diagnostic criteria suggested by the Centers for Disease Control (CDC), within 30 days after surgery

Secondary outcome

- The length of postoperative hospital study

The incidence of 30-day postoperative complications stratified according to the modified Clavien-Dindo classification

Clinical visit and assessment schedule

Baseline at the enrollment, postoperative day 0, postoperative day 1-2, postoperative week 1, postoperative week 2-3, postoperative week 4

			Study period					
		Enrollment	Allocation	Post-allocation				Close-out
	Time point	-2 to 0 day	Operation day (day 0)	Postoperative day 2	Postoperative Week 1	Postoperative Week 2-3	Postoperative Week 4-5	Postoperative week 4-5
Enrollment	Eligibility screen	x						
	Informed consent	x						
	Randomization		x					
	Allocation		x					
Intervention	Experimental intervention		x					
	Control intervention		x					
Assessment	Demographical data	x						
	Medical history	x						
	Nutritional status	x						
	Laboratory	x	x	x				

	examination							
	Parameters of surgical procedure		x					
	Body temperature		x					
	Documentation of SSI				x	x	x	
	Documentation of other complication				x	x	x	
	Length of hospital stay							x
	Readmission rate							x

Original protocol

1. Objectives

The COVER trial aims to investigate the effect of a dual-ring, plastic wound protector in open abdominal surgery. It is designed to test whether the device helps to reduce the overall rate of SSI development within 30 days postoperatively by 40% compared with that of the control group. In particular, the COVER trial includes patients who are undergoing an open abdominal surgery for contaminated or dirty/infected wounds, as well as those undergoing an open abdominal surgery for clean or clean-contaminated wounds, which allows a thorough investigation of the wound protector's effects, depending on the degree of contamination.

2. Background and rationale

Surgical site infection (SSI) is a common postoperative complication that is associated with considerable morbidity and mortality. [1-3] The rate of SSI is estimated to range from approximately 10% to 30% after elective abdominal surgery, depending on the presence of risk factors, type of procedure and degree of endogenous contaminants. [1,4,5] In cases of fecal peritonitis, the SSI rate may reach up to 35~40%. [6,7] Despite organizational, systematic approaches for preventing SSI based on evidence, such as preoperative antibiotic prophylaxis and antiseptic skin cleansing, SSI is still a major problem associated with increased hospital cost, prolonged hospital stays and unsatisfactory quality of life. [8]

The risk of developing an SSI will increase when the surgical incision site is exposed to large amounts of virulent bacteria in a contaminated surgical field. [9] The risk has led to the idea of developing a physical barrier for the wound edge that can hinder direct exposure of the surgical incision edges to the contaminated field. Several devices that are designed for wound edge protection and have a similar design involving a flexible plastic wound cover placed in the laparotomy site are currently on the market. Prospective studies and randomized clinical trials (RCTs) have been conducted to evaluate the effectiveness of plastic wound protectors for reducing the incidence of SSI. [8,10-13]

The largest RCT evaluating the effectiveness of wound protectors in reducing SSI is the Reduction of Surgical Site Infection using a Novel Intervention (ROSSINI) trial. [8] In this study, the drape type of wound protector was compared with standard intraoperative care. The results showed that the use of a wound edge protector during open abdominal surgery did not reduce the rate of SSI. Similarly, RCTs using a drape type of wound protector applied in colorectal surgery reported no benefit of the wound protector in reducing SSI. [10,11] However, several other studies have claimed contrasting results. The BaFO trial, with 608 patients undergoing laparotomy at 16 different medical centers in Germany, demonstrated that the patients who used wound protection drape devices experienced SSI at a lower rate than those who did not. [12] A Japanese single-center RCT with 221 patients enrolled to investigate the effect of a double ring, circular wound protector applied in non-traumatic gastrointestinal surgery also showed that the rate of SSI was significantly lower in the experimental group than in the control group. [13]

The effect of wound protectors in abdominal surgery is still controversial and remains to be elucidated.

A well designed, multicentered RCT evaluating the effect of the dual-ring type of wound protector in open abdominal surgery, particularly for contaminated or dirty infected wounds, has not yet been conducted.

3. Experimental Plan

A. **Trial type** – this is a prospective, multicentered, patient-blinded, randomized controlled trial with two parallel comparison arms.

B. **Trial sites** –Secondary or tertiary hospitals in South Korea

All participating investigators should be educated on the basis of the International Conference on Harmonization (ICH) of Technical Requirements for Registration of Pharmaceuticals for Human Use, which serves as the good clinical practice (GCP) guidelines for this trial. This trial is open for recruiting participating centers.

C. Trial population and eligibility

All gastrointestinal surgical patients undergoing open abdominal surgery, either elective or emergent, will be screened for eligibility.

■ **Inclusion criteria**

(1) Between the ages of 18 and 75 years

(2) Undergoing elective or emergent open abdominal surgery

(3) Undergoing surgery on the stomach, small intestine or colon and rectum

■ **Exclusion criteria**

(1) Presence of concurrent infection in the abdominal wall

(2) Open conversion from laparoscopic surgery

(3) Presence of poor nutritional status indicated by a nutrition risk screening (NRS) 2002 score greater than 3

(4) Undergoing combined hepatobiliopancreatic surgery

(5) Pregnancy or breast feeding

(6) Moderate to severe immunosuppression state (defined as previous organ or bone marrow transplantation, concurrent steroid administration of more than 10mg prednisolone daily or an equivalent dose of any other steroid, concurrent administration of other immunosuppressive or chemotherapeutic agents within the last 2 weeks prior to trial intervention)

D. Recruitment and trial timeline

Patients will be recruited for approximately 48 months. After completion of informed

consent, each subject will be registered. The last follow-up will be made approximately 30 days after the last recruited patient undergoes the trial intervention. An interim analysis is planned when 50% of the enrollment is reached. Depending on the results of the interim analysis, the subsequent research process and timeline can be modified.

E. Randomization and blinding

Stratification will be performed according to the participating center and the type of wound classification. The wound types will be divided into two groups: one group with clean or clean-contaminated wounds and the other group with contaminated or dirty, infected wounds. A web-based patient registry (http://cover.e-trial.co.kr) will be applied to generate the allocation sequence immediately before the beginning of the operation, providing adequate concealment for the allocation sequence. The group allocation and randomization number will be predefined by a biostatistician from the Catholic Medical Center in Seoul, South Korea. A permuted block randomization with the size of two or four will be applied. Participating investigators cannot be blinded to the allocated treatment. However, the patient will be blinded to the trial intervention since they are under general anesthesia once the operation starts. The data manager will also be blinded because there is no direct access to either the trial intervention or the randomization.

F. Screening failure

Registered subjects who are ineligible for the study based on screening assessments will be considered protocol violation due to screening failure and registered as such with reason for failure.

G. Interventions

Preoperative bowel preparation, type of skin preparation and drape, the use of perioperative antibiotics and the details of the surgical procedure will follow the policy of an individual surgeon at each center. The experimental arm will be provided with a circular polyethylene drape (O Trac, Asung Medical, South Korea) to cover the incision site in the abdomen. It is a double-ring type of sterile, cylindrical wound protector consisting of inner and outer rings with a polyethylene sheath. The wound protector is left in situ throughout the operation and is removed immediately before closing the abdominal wall. The method of wound closure and insertion of wound drainage will also follow the policy of an individual surgeon at each center. For the control arm, conventional surgical dressing gauze will be used to protect the incision site during the surgical procedure. There are no differences in surgical techniques, other devices or environments.

H. Risks to the participants

No additional risks to the participants are expected. The circular polyethylene wound protector has established clinical safety and has already been used in clinical applications with the approval of the Korean Medical Device Information and Technology Assistance Center. None of the technical details other than wound protection are affected by the trial.

I. Outcomes

The primary end point is the rate of surgical site infection (SSI), defined by the diagnostic criteria suggested by the Centers for Disease Control (CDC), within 30 days after surgery. SSIs are classified as either superficial incisional, deep incisional or organ/space. [14] The postoperative wound condition will be evaluated at postoperative weeks 1, 2 and 4-5. The secondary end points include the length of postoperative hospital stay, the readmission rate and the rate of surgical complications other than SSI. The incidence of 30-day postoperative complications other than SSI. The incidence of 30-day postoperative complications will be stratified according to the modified Clavien-Dindo classification. [15]

J. Data management and monitoring

A newly developed, web-based, electronic case reporting form (e-CRF) will be used to record data for the included patients. Baseline characteristics, including age, sex, body mass index, American Society of Anesthesiologists score, history of smoking and alcohol consumption, history of previous chemotherapy, radiotherapy, abdominal surgery or steroid or immunosuppressive drug use, history of diabetes or malignancies in the gastrointestinal tract and nutritional status based on the NRS 2002 score, will be collected. Laboratory parameters (white cell counts, C-reactive protein, and albumin levels) will be collected preoperatively, on the operation day and on postoperative day 2, if available. The parameters for the surgical procedures, including operation type (emergent or elective), site of operation (stomach, small bowel or large intestine), level of wound contamination according to CDC classification, method of skin preparation, antibiotic use, operation time, bowel anastomosis and stoma formation, wound closure material, length of skin incision, draining tube for the wound and body temperature during the surgical procedure, will be collected. The surgical wounds are classified as clean, clean-contaminated, contaminated, and dirty wounds, according to the magnitude of the bacterial load. [16] Postoperatively, the surgical wound will be evaluated at postoperative week 1, 2 and 4-5. A photograph of the wound will be taken at each office visit and documented in the eCRF. If SSI is detected, the classification and the postoperative date of diagnosis will be recorded. Bacterial culture of the infected wound will be performed. Postoperative complications according to the modified Clavien-Dindo classification, postoperative length of hospital stay and readmission will be noted. An investigator or research coordinator at each center will enter the data using the eCRF. At the end of the the trial, the study data and personal information of the enrolled patients will be archived for 3 years. The trial data will be monitored by an independent institution (Medical Excellence) in Seoul, Korea. Monitoring will be performed in accordance with ICH-GCP guidelines. [17]

K. Safety evaluation and reporting of adverse events

All adverse events or serious adverse events, occurring from the moment of randomization until the end of the 30-day follow-up, will be recorded and reported by the investigators.

L. Statistical methods

(1) Sample size calculation

The sample size was calculated based on the primary end point of this trial. Previous reports on the incidence of SSI have indicated that the rate of SSI may vary depending on the wound classification, the procedure, the surveillance criteria and the quality of data collection. [18] The incidence of SSI for clean/clean-contaminated wounds has been reported to be as high as 10%. [19] For contaminated wounds, the incidence was approximately 25%. [7,18] For dirty, infected wounds, the incidence may reach up to 40%. [5-7] In this trial, the ratio of operations with clean/clean-contaminated, contaminated and dirty, infected wounds are assumed to be 20:40:40; therefore, the expected incidence of SSI for the control group as 28%. For the experimental group, the incidence of SSI will be decreased by 40%. Thus, the rate of SSI in the experimental group will be approximately 17%. The sample size was determined to achieve a study power of 80%, with 95% two-sided confidence limits. The actual sample size amounts to 434 participants. However, considering a dropout (lost to follow-up, retracted consent or protocol violation) rate of up to 5%, a total of 458 patients, 229 patients in each group, will be enrolled in this study.

(2) Statistical analysis

The statistical analysis will be performed by an independent statistician from the Catholic Medical Center (Seoul, South Korea). The interim and final results will be analyzed mainly for the intention-to-treat population and, additionally, for the per-protocol population. The rate of 30-day postoperative SSI will be evaluated in all patients and analyzed mainly for the intention-to-treat population and, additionally, for the per-protocol population. The rate of 30-day postoperative SSI will be evaluated in all patients and analyzed according to the wound classification (superficial incisional, deep incisional and organ/space SSIs), as defined by the CDC. Pearson's χ^2 test or Fisher's exact test will be used to analyze nominal data; Student's t-test and the Wilcoxon rank-sum test will be used for continuous data. To estimate the independent risk factors for 30-day postoperative SSI, logistic regression analysis will be performed. The statistical analysis will be conducted using SAS V.9.4 (SAS Institute)

M. Ethics, patient withdrawal, and dissemination

Written informed consent will be obtained from each study participant prior to any protocol-related activities in accordance with ethical approval. The principal investigator, surgeon co-investigator, or one of the approved study coordinators must explain orally and in writing the details of the study. Enrolled patients can withdraw their participation at any time if desired. In this case, the patients will have no disadvantages. The investigator will record any patient withdrawal in the CRF. Decisionally-impaired and cognitively impaired persons will not be enrolled, as considered a vulnerable population. Although staff and employees of the participating sites are considered vulnerable populations, they may be eligible to participate in this study. If an employee is a potential candidate for this study, the subject will be informed that participation or refusal to will would not influence grades, employment, or subsequent recommendations. Every effort will be made to prevent

coercion at any time in the process. Students and house staff cannot be asked to participate in research conducted while under the direct supervision of the investigator; therefore, those subjects will not be enrolled.

The investigators and study coordinators are obligated to keep the anonymity and confidentiality of subjects participating in this study. Documents that can identify the subject by name will be tightly regulated to keep confidentiality, except to the extent necessary to allow monitoring and audition by internal and external regulatory authorities.

All information, including name and medical record number, will be stored in a secure database for 3 years. Only the investigator and the designated members of the study team will have access to the separately-stored master list. All electronic records pertaining to the clinical study will be password-protected, and only approved study members listed on the IRB protocol will have password access. Any information about the subject collected on paper will be kept under lock and key in designated place at the corresponding participating site.

Reference

1. De Pastena M, Paiella S, Marchegiani G, et al. Postoperative infections represent a major determinant of outcome after pancreaticoduodenectomy: Results from a high-volume center. Surgery 2017;162:792-801.

2. Badia JM, Casey AL, Petrosillo N, et al. Impact of surgical site infection on healthcare costs and patient outcomes: a systematic review in six European countries. J Hosp Infect 2017;96:1-15.

3. Mujagic E, Marti WR, Coslovsky M, et al. Associations of Hospital Length of Stay with Surgical Site Infections. World J Surg 2018; doi:10.1007/s00268-018-4733-4.

4. Culver DH, Horan TC, Gaynes RP, et al. Surgical wound infection rates by wound class, operative procedure, and patient risk index. National Nosocomial Infections Surveillance System. Am J Med 1991;91:152s-7s.

5. Smith RL, Bohl JK, McElearney ST, et al. Wound infection after elective colorectal resection. Ann Surg 2004;239:599-605; discussion -7.

6. Hernandez K, Ramos E, Seas C, et al. Incidence of and risk factors for surgical-site infections in a Peruvian hospital. Infect Control Hosp Epidemiol 2005;26:473-7.

7. Ruiz-Tovar J, Alonso N, Morales V, et al. Association between Triclosan-Coated Sutures for Abdominal Wall Closure and Incisional Surgical Site Infection after Open Surgery in Patients Presenting with Fecal Peritonitis: A Randomized Clinical Trial. Surg Infect (Larchmt) 2015;16:588-94.

8. Pinkney TD, Calvert M, Bartlett DC, et al. Impact of wound edge protection devices on surgical site infection after laparotomy: multicentre randomised controlled trial (ROSSINI Trial). Bmj 2013;347:f4305.

9. Bruce J, Russell EM, Mollison J, et al. The measurement and monitoring of surgical adverse events. Health Technol Assess 2001;5:1-194.

10. Psaila JV, Wheeler MH, Crosby DL. The role of plastic wound drapes in the prevention of wound infection following abdominal surgery. Br J Surg. 1977;64:729-32.

11. Nystrom PO, Broome A, Hojer H, et al. A controlled trial of a plastic wound ring drape to prevent contamination and infection in colorectal surgery. Dis Colon Rectum. 1984;27:451-3.

12. Mihaljevic AL, Schirren R, Ozer M, et al. Multicenter double-blinded randomized controlled trial of standard abdominal wound edge protection with surgical dressings versus coverage with a sterile circular polyethylene drape for prevention of surgical site infections: a CHIR-Net trial (BaFO; NCT01181206). Ann Surg 2014;260:730-7; discussion 7-9.

13. Horiuchi T, Tanishima H, Tamagawa K, et al. Randomized, controlled investigation of the anti-infective properties of the Alexis retractor/protector of incision sites. J Trauma. 2007;62:212-5. doi: 10.1097/01.ta.0000196704.78785.ae.

14. National Healthcare Safety Network, Centers for Disease Control and Prevention. Surgical site infection (SSI) event. http://www.cdc.gov/nhsn/pdfs/pscmanual/9pscssicurrent.pdf. Published January 2017. Accessed January 25, 2017.

15. Clavien PA, Barkun J, de Oliveira ML, et al. The Clavien-Dindo classification of surgical complications: five-year experience. Ann Surg 2009;250:187-96.

16. Barie PS. Surgical site infections: epidemiology and prevention. Surg Infect (Larchmt) 2002;3 Suppl 1:S9-21.

17. International Conference on Harmonisation of Technical Requirements for Registration of Pharmaceuticals for Human Use (ICH) adopts Consolidated Guideline on Good Clinical Practice in the Conduct of Clinical Trials on Medicinal Products for Human Use. Int Dig Health Legis 1997;48:231-4.

Summary of significant protocol changes

18. Owens CD, Stoessel K. Surgical site infections: epidemiology, microbiology and prevention. J Hosp Infect 2008;70 Suppl 2:3-10.

Summary of Significant Protocol Changes

1. PI Hyung Jin Kim moved to EunPyeong St. Mary's Hospital, the Catholic University of Korea from St. Vincent's Hospital, the Catholic University of Korea in 2019.

    ■ Patients at St. Vincent's Hospital, the Catholic University of Korea were not affected by the transfer of PI Hyung Jin Kim

Final Statistical Analysis Plan

- No significant changes to the original plan are made. All the analyses will be conducted as planned, except for the readmission rate. The re-admission rate is not taken into as a secondary outcome because some patients included in the study are diagnosed with colorectal cancer, which requires readmission for adjuvant or palliative chemotherapy.

- Primary analysis

For the 30-day SSI rate, simple chi-squared tests will be used for unadjusted analyses and a logistic regression model will be used for adjusted analyses. Differences in the baseline demographic and clinical characteristics between the experimental group and the control group are expected to occur at random. Any significant differences found among the demographics, and clinical or surgical characteristics between the two treatment groups will be controlled for in the final analysis to limit potential confounding effects.

Prespecified covariates: Wound types (clean-contaminated / contaminated or dirty), sex, age, nutritional status, the severity of disease, NRS 2002 score, age-adjusted NRS 2002, BMI (Kg/m^2), ASA class, History of diabetes mellitus, smoking, alcohol consumption, the necessity of ICU care, previous history of chemotherapy, previous history of radiation therapy, previous history of abdominal surgery, history of steroid use, history of immunosuppressant; use of antiplatelets or anticoagulants, Gastrointestinal tract cancer history

Surgery timing, surgical site, trauma-related, types of skin preparation, degree of intraperitoneal contamination; use of antibiotics, total surgery time, bowel anastomosis, colostomy formation, use of intraperitoneal drainage, skin suture material, incision length, use of drainage system on the superficial wound, average body temperature during surgery

- Secondary outcome

Postoperative complication rates and length of postoperative hospital stay will be compared between two groups. Chi-square test, Fisher's exact test or the Willcoxon rank sum test will be used.

- Handling missing values

We reported the percentage of missing values of each variable. There were no missing data for the primary

outcome, and the secondary outcomes had missing values of 6.6% for the length of postoperative hospital stay and 4.6% for the rate of postop complication. The confounding variables except for skin prep type (23.6%) had <3% missing values. No imputation of missing data was considered. Only for the multivariable analysis, to minimize sample reduction due to missing covariates, indicator variables were used for missing categorical variables (diabetes milletus 1.8%, colostomy formation 2.4%) or replacing missing values with median (total operation time 2.4%)
