# Supplementary material for: Plastic Wound Protector vs Surgical Gauze for Surgical Site Infection Reduction in Open GI Surgery: A Randomized Clinical Trial
Source: JAMA Surg. 2024 Apr 24;159(7):737–46. doi: 10.1001/jamasurg.2024.0765 (PMC11044008; doi:10.1001/jamasurg.2024.0765)
Supplement: Supplement 2. — eFigure 1. The Dual-Ring Wound Protector (O Trac, Asung Medical, South Korea) Applied to the Incision Site in the Patients of the Experimental Group eFigure 2. The Conventional Surgical Gauze Used to Cover the Incision Site in Patients in the Control Group eTable 1. Sensitivity Analysis for the Rates of Surgical Site Infection eTable 2. The Postoperative Complication Rates and Distribution of Complications by the Clavien-Dindo Classification [file jamasurg-e240765-s002.pdf]

Supplementary Online Content

Yoo N, Mun JY, Kye B-H, et al. Plastic wound protector vs surgical gauze: multicentered randomized controlled trial on surgical site infection reduction in open gastrointestinal tract surgery. *JAMA Surg*. 2024;159(4.4):e240765. doi:10.1001/jamasurg.2024.0765

eFigure 1. The Dual-Ring Wound Protector (O Trac, Asung Medical, South Korea) Applied to the Incision Site in the Patients of the Experimental Group

eFigure 2. The Conventional Surgical Gauze Used to Cover the Incision Site in Patients in the Control Group

eTable 1. Sensitivity Analysis for the Rates of Surgical Site Infection

eTable 2. The Postoperative Complication Rates and Distribution of Complications by the Clavien-Dindo Classification

This supplementary material has been provided by the authors to give readers additional information about their work.

© 2024 Yoo N et al. *JAMA Surg*.

eFigure 1. The dual-ring wound protector (O Trac, Asung Medical, South Korea) applied to the incision site in the patients of the experimental group.

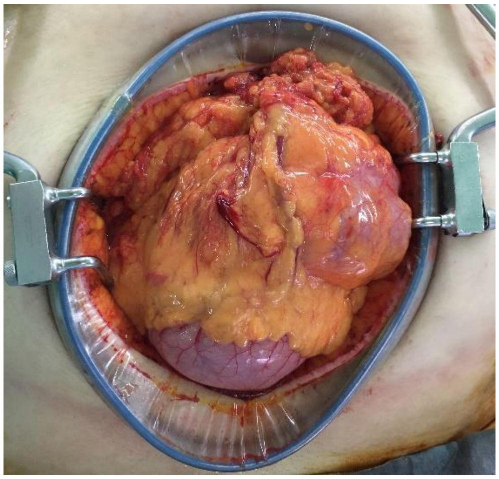

eFigure 2. The conventional surgical gauze used to cover the incision site in patients in the control group.

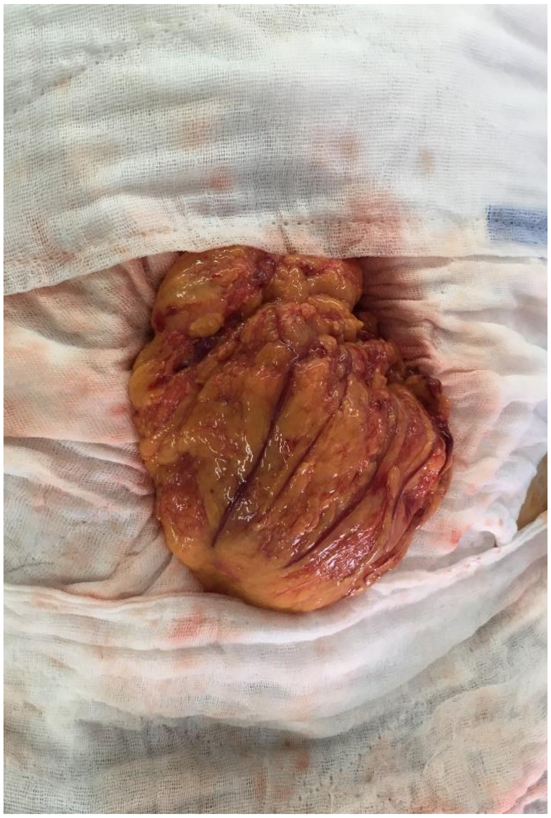

© 2024 Yoo N et al. *JAMA Surg*.

eTable 1. Sensitivity analysis for the rates of surgical site infection

		Total (n=454)	O-trac (n=229)	Gauze (n=225)	Difference (95% CI)	Relative Risk Reduction % (95% CI)	*P*-value	*P*-value*
The overall rate of SSIs		68 (15.0)	25 (10.9)	43 (19.1)	8.19(1.66-14.73)	42.88(9.75-63.84)	0.014	0.015
The rates of SSI by wound types	Clean-contaminated (n=339)	47 (13.9)	18 (10.5)	29 (17.3)	6.74(-0.60-14.07)	39.02(-5.51-64.75)	0.073	
	Contaminated/dirty infected (n=115)	21 (18.3)	7 (12.1)	14 (24.6)	12.49(-1.48-26.46)	50.86(-12.73-78.58)	0.083	
Types of SSI	Superficial	63 (13.9)	23 (10.0)	40 (17.8)	7.73(1.40-14.07)	43.50(8.81-65.00)	0.017	
	Deep	7 (1.5)	3 (1.3)	4 (1.8)	0.47(-1.80-2.74)	26.31(-225.53-83.32)	0.722	

P-values are calculated by the *chi-square* test or Fisher's exact test. **P*-value from the Cochran Mantel Haenszel test stratified by type of wound (randomization stratification factor). The difference is constructed for Gauze minus O-trac, and confidence intervals were using the Wald method.

eTable 2. The postoperative complication rates and distribution of complications by the Clavien-Dindo classification

**Intention-to-treat**		Total (n=458)	O-trac (n=229)	Gauze (n=229)	*P*- value
The overall complication rate		89 (19.4%)	46 (20.1%)	43 (18.8%)	0.414
The Clavien-Dindo classification	Grade I	12 (2.6%)	8 (3.5%)	4 (1.7%)	0.862
	Grade II	37 (8.1%)	18 (7.9%)	19 (8.3%)	
	Grade III	21 (4.6%)	10 (4.4%)	11 (4.8%)	
	Grade IV	12 (2.6%)	7 (3.1%)	5 (2.2%)	
	Grade V	7 (1.5%)	3 (1.3%)	4 (1.7%)	
**Per-protocol**		Total (n=412)	O-trac (n=210)	Gauze (n=202)	*P*- value
The overall complication rate		82 (19.9%)	44 (21.0%)	38 (18.8%)	0.519
The Clavien-Dindo classification	Grade I	12 (2.9%)	8 (3.8%)	4 (2.0%)	0.919
	Grade II	34 (8.3%)	18 (8.6%)	16 (7.9%)	
	Grade III	19 (4.6%)	9 (4.3%)	10 (5.0%)	
	Grade IV	11 (2.7%)	6 (2.9%)	5 (2.5%)	
	Grade V	6 (1.5%)	3 (1.4%)	3 (1.5%)	
