# Supplementary material for: Plastic Wound Protector vs Surgical Gauze for Surgical Site Infection Reduction in Open GI Surgery: A Randomized Clinical Trial
Source: JAMA Surg. 2024 Apr 24;159(7):737–46. doi: 10.1001/jamasurg.2024.0765 (PMC11044008; doi:10.1001/jamasurg.2024.0765)
Supplement: Supplement 3. — Data Sharing Statement [file jamasurg-e240765-s003.pdf]

Data Sharing Statement

Yoo. Plastic Wound Protector vs Surgical Gauze for Surgical Site Infection Reduction in Open GI Surgery. *JAMA Surg*. Published April 24, 2024. doi:10.1001/jamasurg.2024.0765

Data
Data available: Yes
Data types: Deidentified participant data
How to access data: ninayoo1111@gmail.com
When available: With publication

Supporting Documents
Document types: Statistical/analytic code
How to access documents: ninayoo1111@gmail.com
When available: With publication

Additional Information
Who can access the data: researchers whose proposed use of the data has been approved
Types of analyses: metaanalysis
Mechanisms of data availability: with investigator support, without investigator support, after approval of a proposal, and with a signed data access agreement
